# MiR-130a-3p attenuates activation and induces apoptosis of hepatic stellate cells in nonalcoholic fibrosing steatohepatitis by directly targeting TGFBR1 and TGFBR2

**DOI:** 10.1038/cddis.2017.10

**Published:** 2017-05-18

**Authors:** Yang Wang, Jinghua Du, Xuemin Niu, Na Fu, Rongqi Wang, Yuguo Zhang, Suxian Zhao, Dianxing Sun, Yuemin Nan

**Affiliations:** 1 Department of Hepatobiliary Surgery, The Third Hospital of Hebei Medical University, Shijiazhuang 050051, China; 2 Department of Traditional and Western Medical Hepatology, The Third Hospital of Hebei Medical University, Shijiazhuang 050051, China; 3 Department of Liver Disease, Bethune International Peace Hospital, Shijiazhuang 050082, China

## Abstract

Nonalcoholic fibrosing steatohepatitis is a uniform process that occurs throughout nonalcoholic fatty liver disease (NAFLD). MicroRNAs (miRNAs) have been shown to be involved in the biological processes, but the role and molecular mechanism of miRNAs in NAFLD are not entirely clear. In this study, we observed a significant reduction in the expression of miR-130a-3p in livers of a mouse model with fibrosis induced by a methionine–choline-deficient diet, of NAFLD patients, and in activated hepatic stellate cells (HSCs). A dual-luciferase activity assay confirmed that transforming growth factor-beta receptors (TGFBRs) 1 and 2 were both the target genes of miR-130a-3p. The hepatic expression of TGFBR1 and TGFBR2 was significantly increased. Moreover, the overexpression of miR-130a-3p in HSCs inhibited HSC activation and proliferation, concomitant with the decreased expression of TGFBR1, TGFBR2, Smad2, Smad3, matrix metalloproteinase-2 (MMP-2), MMP-9, type I collagen (Col-1), and Col-4. In addition, the overexpression of miR-130a-3p promoted HSC apoptosis by inducing the expression of caspase-dependent apoptosis genes. Transfection with si-TGFBR1 and si-TGFBR2 revealed effects on HSC function that were consistent with those of miR-130a-3p. TGFBR1 and TGFBR2 rescued the miR-130a-3p-mediated reductions in the mRNA and protein expression levels of Smad2, Smad3, Col-1, and Col-4. In conclusion, our findings suggest that miR-130a-3p might play a critical role in negatively regulating HSC activation and proliferation in the progression of nonalcoholic fibrosing steatohepatitis by directly targeting TGFBR1 and TGFBR2 via the TGF-*β*/SMAD signaling pathway.

Nonalcoholic steatohepatitis (NASH), the most extreme form of the nonalcoholic fatty liver disease (NAFLD), is characterized by steatosis, lobular inflammation, and progressive pericellular fibrosis.^1^ Nonalcoholic fibrosing steatohepatitis results from wound healing responses that are attributable to liver injury and induced by free fatty acid accumulation and insulin resistance. Hepatic stellate cells (HSCs) are the major cell type responsible for the progression of nonalcoholic fibrosing steatohepatitis.^2, 3^ Upon liver injury, HSCs undergo an activation process from a fat storing, quiescent phenotype to a highly activated myofibroblastic phenotype, which is characterized by the excessive production of extracellular matrix (ECM), eventually leading to liver fibrosis.^4^ The pathogenesis of nonalcoholic fibrosing steatohepatitis is complicated. There are few effective therapies, and the mechanism of liver fibrosis is poorly understood. However, accumulating studies have demonstrated that microRNAs (miRNAs) are critically involved in the different stages of liver fibrosis, including HSC activation, proliferation, and ECM production and deposition.^5, 6, 7^

miRNAs are small, endogenous, non-coding RNAs that interact with the 3′-untranslated region (UTR) of target mRNAs, resulting in the inhibition of translation or the promotion of mRNA degradation.^8, 9^ miRNAs play important roles in cell proliferation,^10^ development,^11^ differentiation,^12^ and apoptosis,^13, 14, 15^ and they are involved in the development of many chronic liver diseases including viral hepatitis, drug-induced liver injury, and autoimmune liver disease.^16, 17, 18^

Recent research has demonstrated that miRNAs can regulate HSC activation,^5^ proliferation,^6^ and apoptosis^19^ to exert influence on the progression of liver fibrosis. Therefore, we hypothesized that there might also be certain miRNAs that regulate fibrogenesis in NASH by directly targeting downstream molecules. In our study, we evaluated the miRNA profiles of nutritional fibrosing steatohepatitis in mice that were fed a methionine–choline-deficient (MCD) diet.^20, 21, 22^ We found that miR-130a-3p inhibited TGF-*β*/SMAD signaling by directly targeting transforming growth factor-beta receptors (TGFBRs) 1 and 2, which might contribute to the pathogenesis of hepatic fibrosis and provide a potential novel drug target for the treatment of NAFLD.

## Results

### Validation of miRNA expression by quantitative real-time PCR

**Figure 1:**
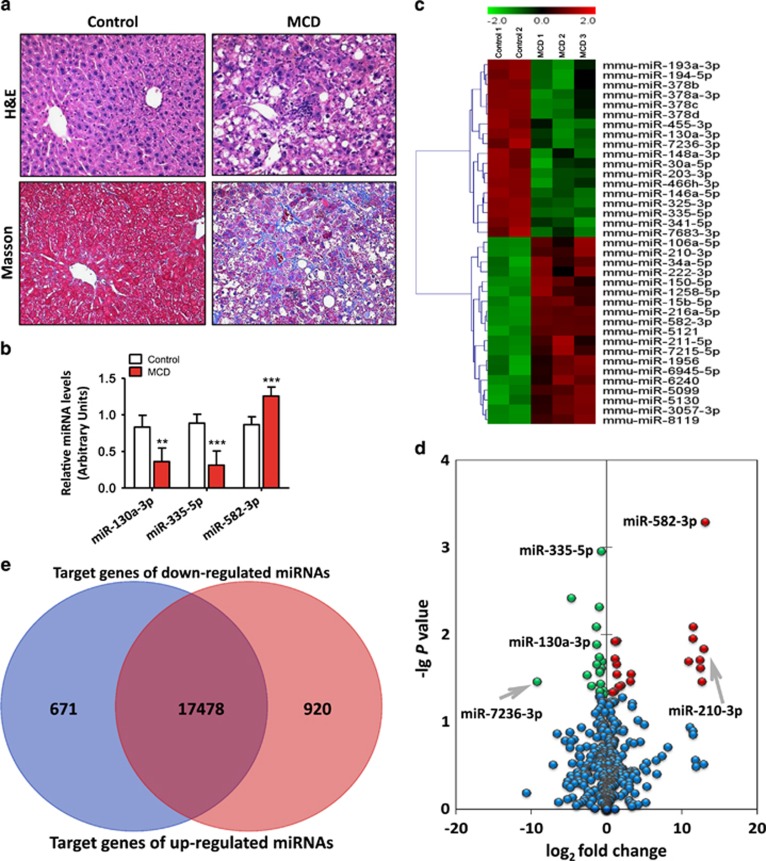
Histopathological changes in liver sections, miRNA microarray data, and validation of the differentially expressed miRNAs in mice. (**a**) Histopathological changes in the liver sections of the mice fed the MCD and control diets. Hematoxylin and eosin staining (up) and Masson’s trichrome staining (down) ( × 200 magnification). (**b**) Validation of microarray data using qRT-PCR. Triplicate assays were done for each RNA sample and the relative amount of each miRNA was normalized to U6 snRNA. Values represent the mean±S.D., **P<0.01, ***P<0.001 compared with control. (**c**) A heatmap of the 37 most differentially regulated miRNAs of mice from the miRNA array of the control and MCD group. (**d**) A volcano plot demonstrating the profiles of the differentially expressed miRNAs for the MCD *versus* control groups. This plot illustrates the fold change (*x* axis) and significance level expressed as the log *P*-value (*y* axis). The green circles represent the miRNAs that were downregulated by the MCD diet compared with the control, and the red circles represent the miRNAs that were upregulated by the MCD diet compared with control. The blue circles indicate the miRNAs that were not significantly expressed. Significance was determined based on a *P*-value cutoff of 0.05 and a 1.3-fold change. (**e**) A Venn diagram illustrating the amount of target genes of the downregulated and upregulated miRNAs as predicted by the following five algorithms: TargetScan, miRanda, miRDB, PITA, and RNA22

The liver sections from mice that were fed the MCD diet exhibited a disordered lobule structure, macrosteatosis in Zone 3, spot or focal hepatocyte necrosis, inflammatory infiltration and perisinusoidal fibrosis (Figure 1a). In line with the microarray assay results, quantitative real-time (qRT) PCR confirmed that miR-582-3p was significantly upregulated, while miR-130a-3p and miR-335-5p were downregulated in fibrosing steatohepatitis. Given these were the most significant downregulated miRNAs in fibrosing steatohepatitis, we chose them as candidates for advanced exploration (Figure 1b).

### Microarray-based gene ontology and pathway analysis of differentially expressed miRNAs

To evaluate the potential involvement of miRNAs in nutritional fibrosing steatohepatitis, we performed miRNA microarray analysis to examine the hepatic miRNA expression profiles in mice with fibrosing steatohepatitis and those with normal histology. There were 19 upregulated and 18 downregulated miRNAs in the livers with fibrosing steatohepatitis compared with the healthy livers (>1.3-fold changes) (Figure 1c). The most upregulated miRNA was miR-582-3p (fold change: 8.72 × 10^3^, *P*-value: 5.14 × 10^-4^), and the most downregulated miRNA was miR-335-5p (fold change: 1.61, *P*-value: 1.10 × 10^-3^; Figure 1d). The target genes of the upregulated and downregulated miRNAs were predicted using five databases: Targetscan (http://www.targetscan.org), miRanda (http://www.microrna.org), miRDB (http://mirdb.org), PITA (http://genie.weizmann.ac.il), and RNA22 (https://cm.jefferson.edu). miRWalk2.0 (http://zmf.umm.uni-heidelberg.de/mirwalk2) was used to help develop the strategy for target gene prediction and the subsequent bioinformatics analysis. A total of 19 069 target genes were predicted, 671 and 920 of which were based on only the down- and upregulated genes, respectively, and 17 478 genes formed the overlap of all of the genes potentially targeted by both the downregulated and upregulated miRNAs (Supplementary Data 1 and 2), as predicted by the algorithms above (Figure 1e).

**Figure 2:**
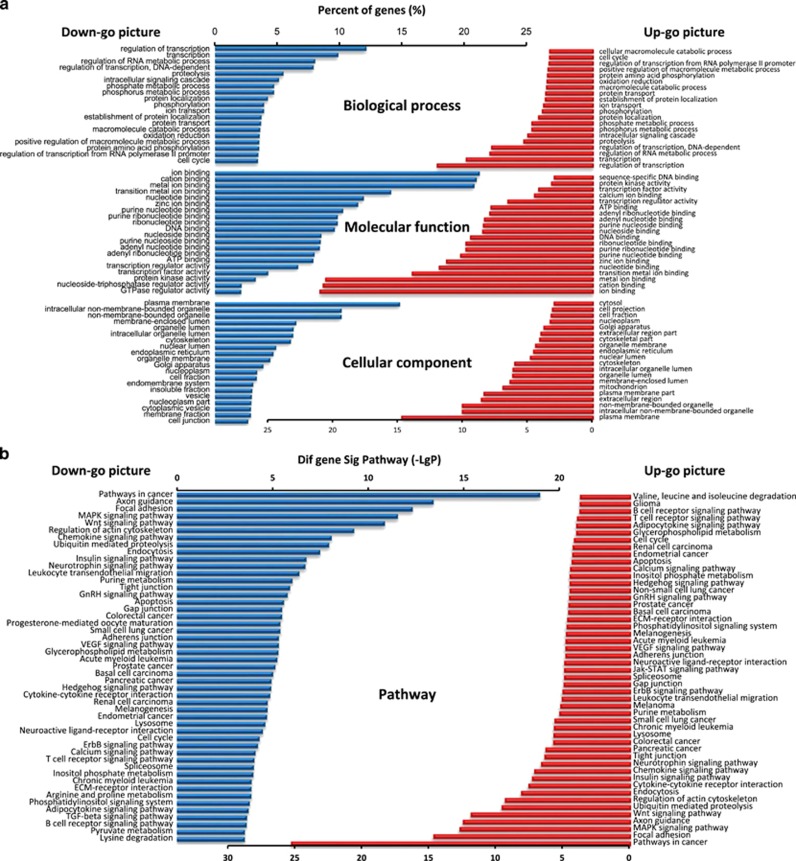
Bioinformatics analysis of the predicted targets of all of the differentially regulated miRNAs. **(a**) GO category classification based on the predicted target genes of all of the differentially regulated miRNAs. The chart includes the GOs targeted by the underexpressed (left side) and overexpressed miRNAs (right side). The percentage of genes represents the number of genes annotated by the gene ontology database to the GO terms. Only the top 20 GO categories are shown for every ontology. The vertical axis is the GO category, and the horizontal axis is the enrichment of GO. **(b)** Pathway analysis was used to place the differentially expressed genes according to KEGG. The chart includes the pathways targeted by the underexpressed (left side) and overexpressed miRNAs (right side). Fisher’s exact tests were used to identify the pathways. Only the top 50 pathways with *P*<0.05 are shown. The vertical axis represents the pathway category, and the horizontal axis represents the value of –log *P*

The gene ontology (GO) database (http://www.geneontology.org) was then used to perform GO analyses of the target genes. Approximately 19 069 predicted genes were classified according to GO term, including biological process, molecular function, and cellular component. The downregulated and upregulated genes were individually analyzed, and only the top 20 GO categories are shown in the differential ontologies (Figure 2a). Fisher's exact test was used to calculate the *P*-value, the false discovery rate (FDR) and perform multiple comparisons (*P*<0.001, FDR<0.01).

We use either the *χ*^2^-test or Fisher’s exact test to perform pathway enrichment analyses based on the KEGG pathway analysis. Pathways with more annotations than anticipated among the differentially expressed genes (*P*<0.001) were considered significantly enriched. Figure 2b displays the top 50 pathways that were over-represented. The downregulated genes primarily participate in the Wnt signaling pathway, the insulin signaling pathway, apoptosis, ECM–receptor interactions, and the TGF-*β*/SMAD signaling pathway. Many of the upregulated genes are linked to the Wnt signaling pathway, cytokine–cytokine receptor interactions, the insulin signaling pathway, ECM–receptor interactions, and apoptosis. Therefore, the GO term and KEGG pathway annotations for the predicted miRNA targets reflected the probable roles of these differentially expressed miRNAs during nonalcoholic fibrosing steatohepatitis.

### Validation of miR-130a-3p expression in an MCD mouse model and in patients

**Figure 3:**
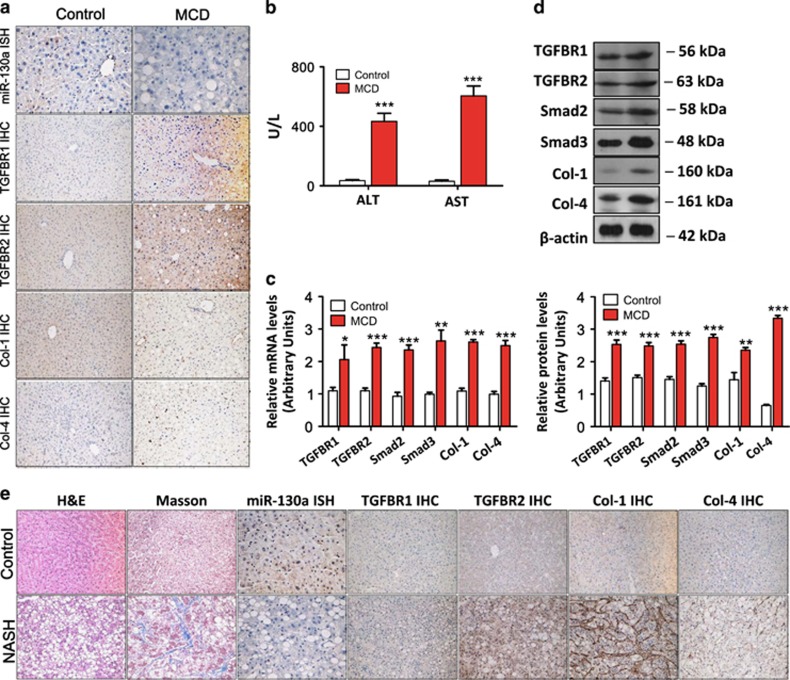
Validation of the differential expression of miR-130a-3p in the livers of mice and patients. **(a**) miR-130a-3p expression was decreased, and the expression of TGFBR1, TGFBR2, Col-1, and Col-4 was increased in the liver tissues of the MCD-fed mice. TGFBR1, TGFBR2, Col-1, and Col-4 expression was detected by IHC ( × 200 magnification), and miR-130a-3p expression was detected using an ISH ( × 400 magnification) assay. Positive staining is indicated by a brown color. **(b)** Effects of the MCD diet on serum ALT and AST levels. Values represent the mean±S.D., ***P<0.001 compared with control. **(c)** Hepatic mRNA and **(d)** protein expression of TGFBR1, TGFBR2, Smad2, Smad3, Col-1, and Col-4 were upregulated in the MCD-fed mice compared with the control mice. *β*-actin was used as a loading control. Values represent the mean±S.D., *P<0.05, **P<0.01, ***P<0.001 compared with control. **(e)** Histopathological changes were evaluated with hematoxylin and eosin staining, and Masson’s trichrome staining. miR-130a-3p expression was decreased, and TGFBR1, TGFBR2, Col-1, and Col-4 levels were increased in the liver tissues of patients with NASH-related liver fibrosis. The expression of miR-130a-3p was detected by ISH ( × 400 magnification), and TGFBR1, TGFBR2, Col-1, and Col-4 were detected using an IHC ( × 200 magnification) assay. Positive staining is indicated by a brown color

According to the microarray data, miR-130a-3p was significantly downregulated in the mouse livers with fibrosing steatohepatitis, which was validated by *in situ* hybridization (ISH) and qRT-PCR. Compared with the control, hepatic miR-130a-3p was significantly downregulated in the MCD diet-fed mice (Figure 3a), which was consistent with the microarray data. Additionally, these changes were accompanied by a parallel increase in the expression of hepatic TGFBR1, TGFBR2, Col-1 and Col-4 (Figure 3a), as assessed by immunohistochemistry (IHC), as well as significantly higher serum ALT and AST levels (*P*<0.001), compared with the control diet-fed mice (Figure 3b). The hepatic mRNA and protein levels of TGFBR1, TGFBR2, Smad2, Smad3, Col-1, and Col-4 were upregulated in the MCD-fed mice compared with the control (Figures 3c and d). The increase in miR-130a-3p was also confirmed in the liver sections of patients with NASH-related fibrosis. The progression of liver injury and the development of fibrosis were assessed based on different pathological changes, increasing fibrosis scores, and increased ALT and AST levels (Table 1). miR-130a-3p expression was dramatically decreased in the liver tissues of patients with NASH-related liver fibrosis. Moreover, the TGFBR1, TGFBR2, Col-1, and Col-4 levels were increased, which coincides with the MCD mouse model as shown in Figure 3e.

### Effects of miR-130a-3p on HSC activation and collagen deposition

**Figure 4:**
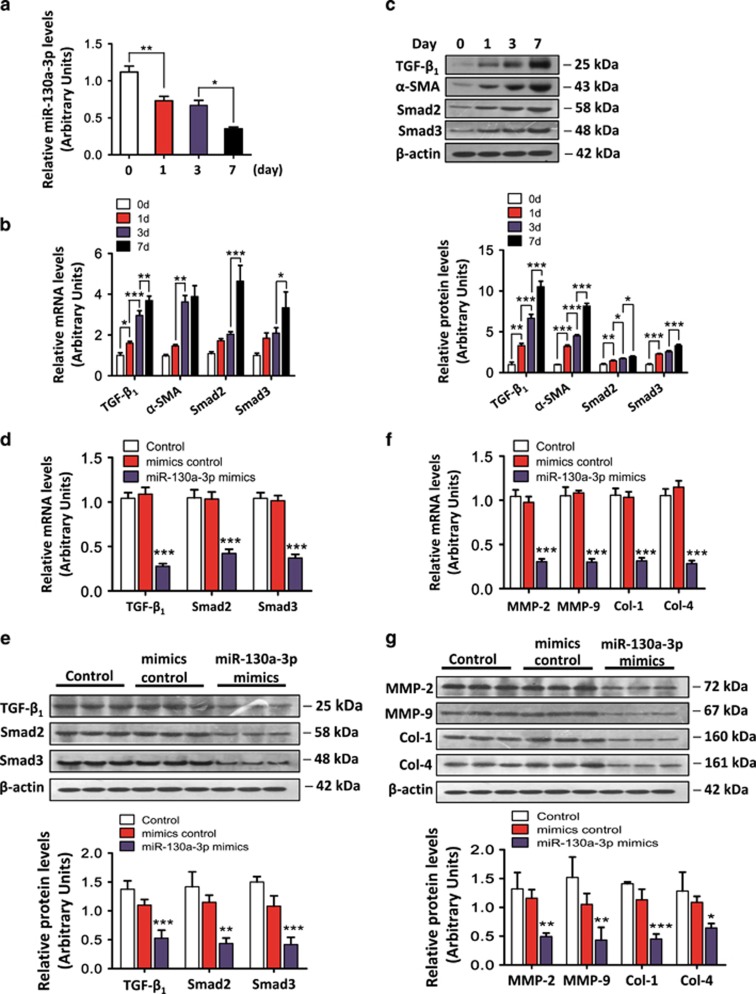
miR-130a-3p expression was downregulated and regulated the downstream expression of genes of the TGF-β/SMAD signaling pathway in activated HSCs. (**a**) miR-130a-3p expression was examined by real-time qRT-PCR during HSC activation. Values represent the mean±S.D., (*P<0.05, **P<0.01). (**b**) The hepatic mRNA and (**c**) protein expression of TGF-*β*_1_, *α*-SMA, Smad2, and Smad3 were increased during the process of HSC activation. *β*-actin was used as a loading control. Values represent the mean±S.D. (*P<0.05, **P<0.01, ***P<0.001). (**d**) HSC-T6 cells were transfected with miR-130a-3p mimics or mimics control for 48 h. The mRNA and protein (**e**) levels of TGF-*β*_1_, Smad2, and Smad3 were analyzed by qRT-PCR and western blotting, respectively. Mimics control is the negative control of mimics, and control is the blank control in this group. The result showed that there was no significant difference between control and mimics control. *β*-actin was used as a loading control. Values represent the mean±S.D., **P<0.01, ***P<0.001 compared with control. (**f**) The mRNA and protein (**g**) levels of MMP-2, MMP-9, Col-1, and Col-4 were analyzed by qRT-PCR and western blotting, respectively. *β*-actin was used as a loading control. Values represent the mean±S.D., *P<0.05, **P<0.01, ***P<0.001 compared with control

A significant downregulation of miR-130a-3p expression was observed in the activated HSCs (Figure 4a). The mRNA and protein expression levels of TGF-*β*_1_ and *α*-SMA, which are markers of HSC activation, were significantly increased in the activated HSCs *in vitro*. Moreover, the mRNA and protein expression levels of Smad2 and Smad3, which are involved in the process of HSC activation, were significantly increased in the activated HSCs (Figures 4b and c). To clarify the roles of miR-130a-3p in HSC activation and collagen deposition, we transfected the HSCs with miR-130a-3p mimics or mimics control. The overexpression of miR-130a-3p significantly suppressed the mRNA and protein levels of TGF-*β*_1_, Smad2, and Smad3 (Figures 4d and e). As for collagen deposition, the mRNA and protein expression levels of matrix metalloproteinase-2 (MMP-2), MMP-9, Col-1, and Col-4 were decreased by transfection with miR-130a-3p mimics compared with the controls, indicating that the overexpression of miR-130a-3p could inhibit HSC activation and collagen deposition via suppressing the TGF-*β*/SMAD signaling pathway, as shown in Figures 4f and g.

### Overexpression of miR-130a-3p suppresses proliferation and induces apoptosis in HSCs

**Figure 5:**
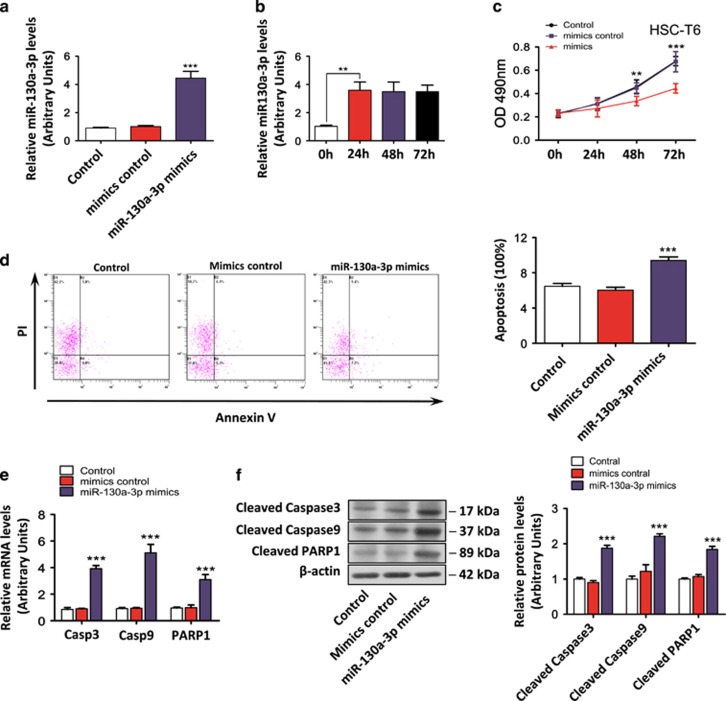
miR-130a-3p inhibited cell proliferation and growth, and induced apoptosis in activated HSCs. **(a)** The miR-130a-3p mimics regulated the expression of miR-130a-3p. qRT-PCR validated the expression of miR-130a-3p after transfection with the miR-130a-3p mimics. The miR-130a-3p mimics significantly upregulated the expression of miR-130a-3p. Values represent the mean±S.D., ***P<0.001 compared with control. **(b)** miR-130a-3p expression was examined by real-time qRT-PCR at 0, 24, 48, 72 h after transfection with the miR-130a-3p mimics. Values represent the mean±S.D. (**P<0.01). (**c**) miR-130a-3p inhibited HSC-T6 cell growth as determined by an MTT assay. Values represent the mean±S.D., **P<0.01, ***P<0.001 compared with control. (**d**) Annexin V/PI combined with flow cytometry. (**e**) The mRNA expression of caspase-3, caspase-9, and PARP1 and (**f**) the protein expression of cleaved caspase-3, caspase-9, and PARP1 were increased after transfection with the miR-130a-3p mimics. *β*-actin was used as a loading control. Values represent the mean±S.D., ***P<0.001 compared with control

To further investigate the effect of miR-130a-3p on the proliferation of HSCs, we first transfected HSC-T6 cells with miR-130a-3p mimics or mimics control and then used an MTT [3-(4,5-dimethylthiazol-2-yl)-2,5-diphenyltetrazolium bromide] assay to measure the proliferation of the HSCs. qRT-PCR showed that miR-130a-3p expression was significantly upregulated after transfection with the miR-130a-3p mimics compared with the controls (Figure 5a). Compared with the baseline, the expression of miR-130a-3p was significantly upregulated 24, 48, and 72 h after transfection (Figure 5b). As shown in Figure 5c, the overexpression of miR-130a-3p inhibited the proliferation of HSCs compared with the controls.

To ascertain whether the observed suppressive effect on cell growth by miR-130a-3p was due to the induction of apoptosis, cellular apoptosis was evaluated by Annexin V/propidium iodide (PI) double staining and flow cytometry. Ectopic expression of the miR-130a-3p mimics in the HSC-T6 cells caused a significant increase in the number of apoptotic cells (*P*<0.05, Figure 5d). Moreover, as indicators of apoptosis, the mRNA expression levels of caspase-3, caspase-9, and poly(ADP-Ribose) polymerase 1 (PARP1) were significantly increased compared with the controls (Figure 5e, *P*<0.001). Consistent with the mRNA expression levels, the protein levels of the active forms of critical apoptosis genes including cleaved caspase-3, caspase-9, and PARP1 were enhanced in the HSC-T6 cells transfected with miR-130a-3p mimics compared with the controls, as assessed by western blotting (Figure 5f, *P*<0.001). These findings indicate that miR-130a-3p can induce cell death and promote subsequent proliferative activity in HSCs.

### miR-130a-3p directly acts on the 3′-UTRs of TGFBR1 and TGFBR2 mRNA

**Figure 6:**
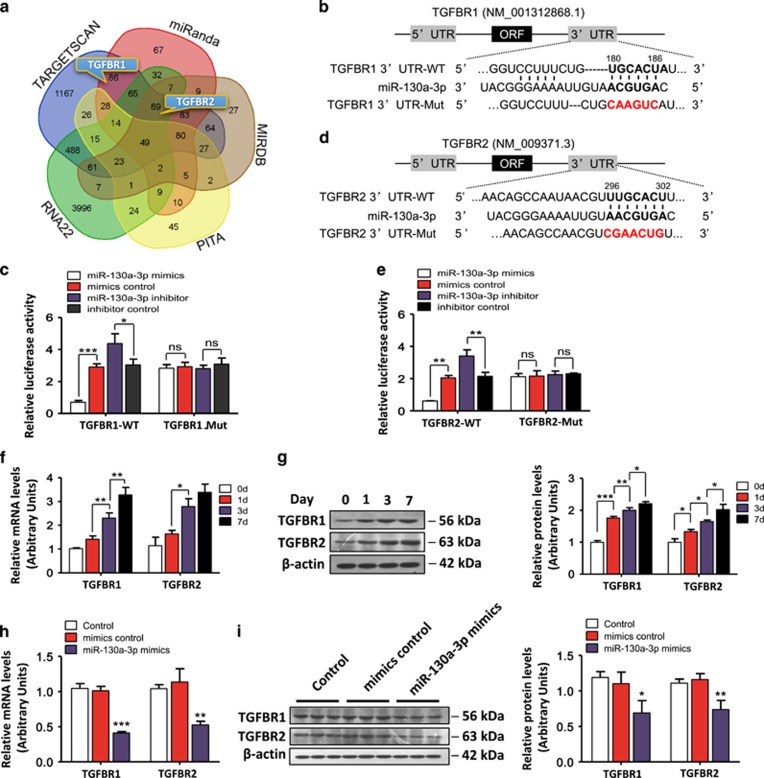
Validation of TGFBR1 and TGFBR2 as target genes of miR-130a-3p. (**a)** A Venn diagram showing the overlap of the genes potentially targeted by miR-130a-3p as predicted by the following five algorithms: TargetScan, miRanda, miRDB, PITA, and RNA22. (**b**, **d**) The potential binding sites for miR-130a-3p on the 3′-UTRs of TGFBR1 and TGFBR2. (**c**, **e**) HEK293T cells were transfected with a luciferase reporter vector containing either the wild-type or mutant form of the 3′-UTRs of TGFBR1 and TGFBR2 in the presence of either the miR-130a-3p mimics, the mimics control, the miR-130a-3p inhibitor or the inhibitor control. The cells were assessed for luciferase reporter activity 48 h post transfection. Values represent the mean±S.D. (*P<0.05, **P<0.01, ***P<0.001). (**f**) The hepatic mRNA and (**g**) protein expression levels of TGFBR1 and TGFBR2 were increased during the process of HSC activation. *β*-actin was used as a loading control. Values represent the mean±S.D. (*P<0.05; **P<0.01; ***P<0.001). (**h**) The HSC-T6 cells were transfected with the miR-130a-3p mimics or mimics control for 48 h. The mRNA and **(i)** protein expression levels of TGFBR1 and TGFBR2 were reduced by the miR-130a-3p mimics in the HSC-T6 cells. *β*-actin was used as a loading control. Values represent the mean±S.D., *P<0.05, **P<0.01, ***P<0.001 compared with control. NS, not significant

To identify the target genes through which miR-130a-3p exerts its effects on HSCs, we used bioinformatics analysis to predict and rank the putative miR-130a-3p target genes related to hepatic fibrogenesis. The available miRNA target prediction programs Targetscan, miRanda, miRDB, PITA, and RNA22 (Supplementary Data 3) indicated that TGFBR1 and TGFBR2 were putative targets of miR-130a-3p (Figure 6a). We found that the 3′-UTRs of TGFBR1 (Figure 6b) and TGFBR2 (Figure 6d) contained putative binding sites for miR-130a-3p. To verify whether miR-130a-3p directly binds to the 3′-UTRs of these candidate genes and causes translational inhibition, we performed dual-luciferase reporter assays using HEK293T cells. The miR-130a-3p mimics significantly decreased the luciferase activity of the TGFBR1-3′-UTR-dependent and TGFBR2-3′-UTR-dependent reporters, but it did not affect the luciferase activity of the mutant reporter. However, the mimics control had no effect on either wild-type (WT) or mutant reporter luciferase activity (Figures 6c and e), suggesting that miR-130a-3p directly acts on the 3′-UTRs of TGFBR1 and TGFBR2 mRNA.

### miR-130a-3p downregulates the expression of TGFBR1 and TGFBR2

We next assessed whether the TGFBR1 and TGFBR2 genes are regulated following miR-130a-3p dysregulation, and consistent results were observed *in vitro*. The miR-130a-3p target genes TGFBR1 and TGFBR2 were significantly enriched during HSC activation (Figures 6f and g). In the HSC-T6 cells, both the mRNA and protein levels of TGFBR1 and TGFBR2 were decreased after transfection with miR-130a-3p mimics compared with controls (Figures 6h and i). These results suggest that miR-130a-3p regulates TGFBR1 and TGFBR2 gene expression at both of the transcriptional and posttranscriptional levels.

### Knockdown of TGFBR1 or TGFBR2 inhibits the expression of the downstream genes of TGF-*β*_1_

**Figure 7:**
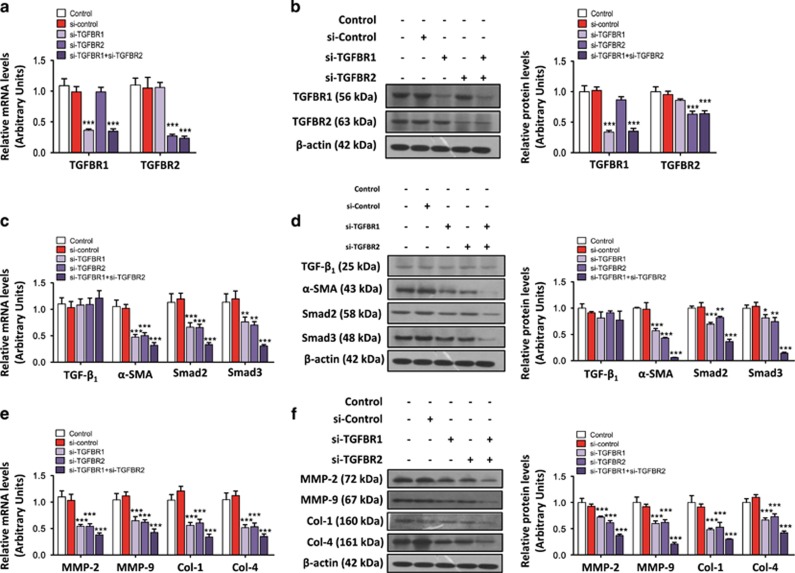
Knockdown of either TGFBR1 or TGFBR2 inhibited the TGF-*β*_/_SMAD signaling pathway and reduced HSC activation. (**a**) Knockdown of TGFBR1 and/or TGFBR2 inhibited the mRNA and protein expression levels (**b**) of TGFBR1 and TGFBR2. β-actin was used as a loading control. Values represent the mean ±SD, ***P < 0.001 compared with control. (**c**) Knockdown of TGFBR1 and/or TGFBR2 inhibited the mRNA and protein expression levels (**d**) of *α*-SMA, Smad2, and Smad3. β-actin was used as a loading control. Values represent the mean ±SD, *P<0.05, **P<0.01, ***P<0.001 compared with control. (**e**) Knockdown of TGFBR1 and/or TGFBR2 inhibited the mRNA and protein levels (**f**) of MMP-2, MMP-9, Col-1, and Col-4. *β*-actin was used as a loading control. Values represent the mean±S.D., ***P<0.001 compared with control

To characterize the effects of TGFBR1 and TGFBR2 on liver fibrosis, we knocked down the expression of TGFBR1 and TGFBR2 in HSC-T6 cells using siRNA transfection. The mRNA and protein levels were significantly decreased after siRNA transfection (Figures 7a and b), and knockdown of TGFBR1 and TGFBR2 significantly reduced the mRNA and protein expression levels of the HSC activation markers *α*-SMA, Smad2, and Smad3 (Figures 7c and d) and the collagen deposition markers MMP-2, MMP-9, Col-1, and Col-4 (Figures 7e and f). These results suggest that knockdown of either TGFBR1 or TGFBR2 inhibits fibrogenesis genes by blocking the TGF-*β*/SMAD signaling pathway.

### TGFBR1 and/or TGFBR2 rescue miR-130a-3p-impaired activation and collagen deposition in HSCs

**Figure 8:**
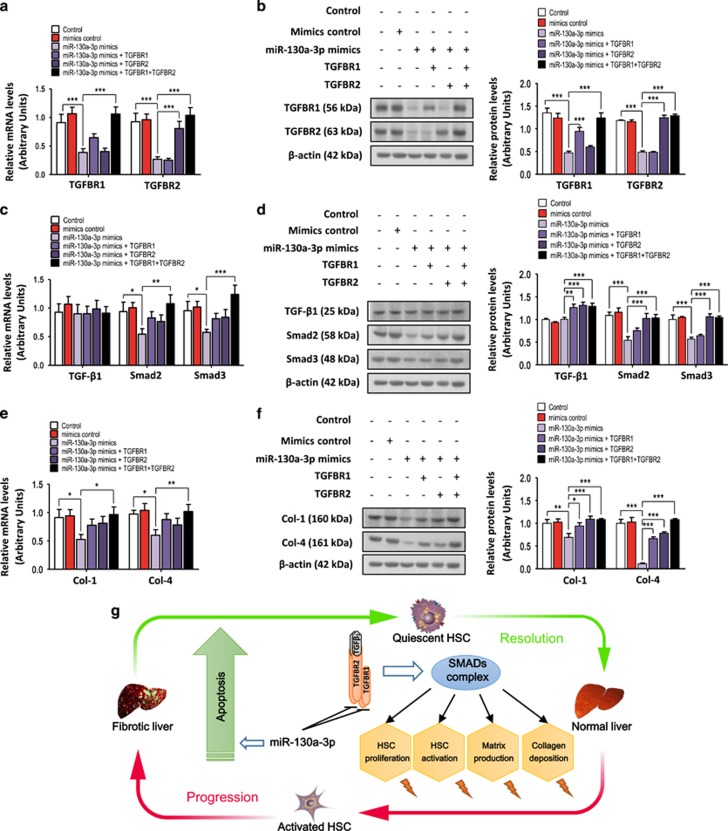
Overexpression of TGFBR1 and TGFBR2 rescued miR-130a-3p-impaired proliferation and cell growth in HSCs. The HSC-T6 cells were transduced with the miR-130a-3p mimics and the TGFBR1-expressing and TGFBR2-expressing vectors. The rescue efficiency of TGFBR1 or TGFBR2 in the HSC-T6 cells was confirmed by (**a**) RT-PCR and (**b**) western blotting. *β*-actin was used as a loading control. Values represent the mean±S.D. (***P<0.001). Overexpression of TGFBR1 and TGFBR2 rescued the **(c)** mRNA and (**d**) protein expression of Smad2 and Smad3. *β*-actin was used as a loading control. Values represent the mean±S.D. (*P<0.05, **P<0.01, ***P<0.001). Overexpression of TGFBR1 and TGFBR2 rescued the (**e**) mRNA and (**f**) protein expression of Col-1 and Col-4. *β*-actin was used as a loading control. Values represent the mean±S.D. (*P<0.05, **P<0.01, ***P<0.001). (**g**) A schematic diagram highlighting the regulation of HSCs by miR-130a-3p during the genesis and resolution of liver fibrosis

To assess whether TGFBR1 and TGFBR2 are key functional targets of miR-130a-3p in HSCs, we performed rescue experiments (Figures 8a and b). TGFBR1 and TGFBR2 rescued the miR-130a-3p-mediated reduction in the mRNA and protein expression levels of Smad2 and Smad3 (Figures 8c and d). Meanwhile, Col-1 and Col-4 expression in the cells was also slightly inhibited by miR-130a-3p, and re-expression of TGFBR1 and TGFBR2 rescued this inhibition (Figures 8e and f). Therefore, the inhibition of activation and collagen deposition by miR-130a-3p was reversed in the cells co-expressing TGFBR1 and TGFBR2. Apparently, additional amounts of TGFBR1 and TGFBR2 above the endogenous levels were not needed and not able to further promote activation and collagen deposition in HSCs. Overall, our results indicate that TGFBR1 and TGFBR2 are functionally relevant for the miR-130a-3p-mediated regulation of HSC activation and collagen deposition.

## Discussion

Nonalcoholic fibrosing steatohepatitis is a multistep process, and its pathogenesis has not yet been clearly defined. In the present study, we used an MCD diet to induce nonalcoholic fibrosing steatohepatitis, which is an ideal model for studying the mechanisms driving NASH-related inflammation and fibrosis.^23^ The biochemical and histopathological results were clearly indicative of the stage of the mouse model and patients with advanced NASH. From the miRNA microarray data, we identified the hepatic miRNAs profiles of nonalcoholic fibrosing steatohepatitis in mice. The target genes of 19 upregulated and 18 downregulated miRNAs were predicted and then used to perform GO analysis and KEGG signaling pathway analysis, respectively. The target genes of the upregulated miRNAs were mainly related to the mitochondrion, DNA binding, transcription, the Wnt signaling pathway, and MAPK signaling and the target genes of the downregulated miRNAs were related to the cytoskeleton, ion binding, regulation of RNA metabolic processes, the Wnt signaling pathway, and the TGF-*β*/SMAD signaling pathway. It has been proposed that differentially expressed miRNAs might be involved in the development of nonalcoholic fibrotic steatohepatitis through the mediation of target genes and related signaling pathways.

To validate the miRNA expression profile, we jointly performed an miRNA microarray assay and stem-loop real-time PCR to examine the expression of aberrantly expressed miRNA. We demonstrated for the first time that hepatic miR-130a-3p expression was one of the most significantly downregulated miRNAs in the fibrotic steatohepatitis mice compared with the control mice. What is more, we further confirm the result in liver sections of both MCD mouse model and NAFLD patients by ISH and IHC. In agreement with our findings, various miRNAs have been suggested to participate in the progression of liver fibrosis through different mechanisms. miR-146a-5p modulates the activation and proliferation of HSCs in the progression of nonalcoholic fibrosing steatohepatitis by modulating the targeted genes of Wnt1, Wnt5a, and related signal transduction pathways.^24^ miR-378 inhibits HSC activation and liver fibrosis by suppressing Gli3 expression.^25^ miR-132 appears to inhibit HSC activation by negatively regulating MeCP2, which leads to the silencing of peroxisome proliferator-activated receptor-*γ* (PPAR-*γ*).^26^ The overexpression of miR-122 in HSCs results in significant inhibition of the production of mature Col-1.^27^ Similarly, a downregulation of miR-130a-3p has been observed during the activation process of HSCs compared with their quiescent phenotype. Therefore, these data suggest that miR-130-3p might play an essential role in the progression of nonalcoholic fibrosing steatohepatitis, and its suppression may be attendant with HSC activation.

HSC activation is the key pathophysiological feature of NASH. Here, we detected a significant elevation of the specific markers TGF-*β*_1_ and *α*-SMA during the process of HSC activation. Interestingly, overexpression of miR-130a-3p inhibited the expression of TGF-*β*_1_ and *α*-SMA. TGF-*β*_1_ is a central regulator of liver fibrosis, and the TGF-*β*/SMAD signaling pathway is probably the most prominent direct inducer of HSC activation and collagen transcription.^28^ Smad proteins transduce signals from TGF-*β*_1_ to regulate cell proliferation, differentiation, and death. Smad2 and Smad3 are two relevant receptor-regulated Smads (r-Smad) that are directly phosphorylated by TGF-*β*_1_ receptors through their intracellular kinase domain, leading to r-Smad activation.^29^ Knockdown of Smad3 with RNAi suppresses TGF-*β*_1_-induced collagen matrix expression. In response to TGF-*β*_1_, Smad2 and Smad3 might work in a reciprocal manner during ECM production and tissues fibrosis. According to these results, the components of the TGF-*β*/SMAD signaling pathway are probably significantly prominent inducers of collagen transcription in HSCs.^30^

Our study demonstrates that the overexpression of miR-130a-3p inhibits the expression of the profibrotic genes Col-1 and Col-4, and the expression of MMP-2 and MMP-9. MMPs are a family of primary enzymes that are implicated in ECM degradation.^31^ Dysregulation of MMP activity results in liver damage and even fibrosis.^32^ MMP-2 and MMP-9 are key factors in the MMP-mediated proteolytic degradation of the ECM and are stimulated by TGF-*β*_1_ during fibrogenesis.^33^ The synthesis and deposition of collagen play a pivotal role in liver repair and remodeling during the progression of liver fibrosis.^30^ In line with this, we suggest that overexpression of miR-130a-3p might repress collagen synthesis and deposition in fibrosing steatohepatitis.

In addition to blocking the deposition of collagen, the overexpression of miR-130a-3p significantly inhibited HSC proliferation. The induction of apoptosis in HSCs by miR-130a-3p was also detected concurrently with the inhibition of cellular proliferation, whereby apoptosis was executed by the regulation of caspase-3, caspase-9, and PARP. In fact, it has been demonstrated that HSC apoptosis is an essential feature of NASH-related liver fibrosis and may be correlated with NAFLD severity.^34^ Increased expression of the miR-130a-3p-activated initiator caspase-9 and effector caspase-3 prompts the proteolytic cleavage of PARP, leading to cellular disassembly and apoptosis. Much attention has been focused on the process of activation of HSC apoptosis because stimulation of this process *in vivo* promotes the acceleration of the resolution of liver fibrosis.^35^ Here, we demonstrated that transfection with miR-130a-3p significantly increased the susceptibility of HSCs to caspase-mediated apoptosis, which was consistent with the flow cytometry results. Therefore, this may add further evidence to the overall antifibrotic effect of miR-130a-3p. The induction of apoptosis in activated HSCs is one of the effective strategies for reversing or decreasing fibrotic changes in the liver.^36^ In line with our results, miR-130a and miR-130b enhance the activation of hepatic stellate cells by suppressing PPAR-*γ* expression.^37^ These findings imply that miR-130a-3p might negatively regulate liver fibrogenesis by inhibiting HSC activation and collagen synthesis.

The role of miRNA in the fibrogenic process of steatohepatitis depends on the effects of its target genes on liver fibrosis. In our study, the potential target genes of miR-130a-3p were searched jointly using the TargetScan, miRanda, miRDB, PITA, and RNA22 databases. In fact, one single miRNA can regulate many targeted genes (Figure 6a). TGFBR1 and TGFBR2 were predicted to be the direct targets of miR-130a-3p by bioinformatics analysis and were confirmed using a dual-luciferase reporter assay. Additionally, overexpression of miR-130a-3p in HSCs resulted in the downregulation of TGFBR1 and TGFBR2 expression at both the mRNA and protein level, confirming that miR-130a-3p regulates TGFBR1 and TGFBR2 expression at both of the transcriptional and posttranscriptional level. The activation of TGF-*β*/SMAD signaling via TGFBR1/TGFBR2 complexes is tightly regulated at the receptor level and promotes hepatic fibrosis by enhancing the activation and survival of HSCs, and this is one of the major signal transduction pathways associated with hepatic fibrogenesis.^30, 38^ Because the roles of the target genes TGFBR1 and TGFBR2 in NAFLD disease pathways are so prominent, we preferred to study miR-130a-3p but not the other miRNAs that were even more upregulated or downregulated like miR-582-3p or miR-335-5p, as shown in the volcano plot. Although much remains to be learned, our current knowledge suggests important roles for posttranslational modifications of the receptors in defining the levels of functional TGFBRs on the cell surface. To further determine whether TGFBR1 and TGFBR2 are the primary targets mediating the activity of miR-130a-3p, we used a combined loss-of-function approach to characterize the functionality of TGFBR1 and TGFBR2 in fibrogenesis. Our results showed that knockdown of either TGFBR1 or TGFBR2 mimicked the roles of miR-130a-3p, further indicating that TGFBR1 and TGFBR2 are the primary functional targets of miR-130a-3p in nonalcoholic fibrosing steatohepatitis.

Although inhibition of the kinase activity of TGFBR is clearly the most recurrent strategy currently used in preclinical and clinical therapies related to this cytokine, no significant studies involving liver fibrosis have been performed. However, a large number of inhibitors of TGFBR1 kinase have been designed and preclinically tested in various fibrosis-related diseases, and these may eventually be used to treat liver fibrosis. These may affect the different pools of TGF-*β*_1_ receptors at different spatial locations and, consequently, lead to different outcomes. Here, our data showed that suppression of TGFBR1 and TGFBR2 by miR-130a-3p resulted in decreased HSC activation and collagen deposition, and induced apoptosis in HSCs. The downregulation of TGFBR by another miRNA also had antifibrotic effects on the liver^39^ and pulmonary fibrosis.^40^ On the basis of these observations, we suggest that the miRNA-mediated diminishment of TGFBR decreases its overall level, which in turn overrides the spatial or temporal regulation of TGF-*β*/SMAD signaling by different TGFBR regulators. Similarly, the overexpression of TGFBR1 and TGFBR2 could also bypass the specific regulation of this molecule both spatially and temporally.

In summary, we identified hepatic miRNAs and evaluated their expression patterns in nonalcoholic fibrosing steatohepatitis induced by an MCD diet using microarray analysis. Among the validated miRNAs, miR-130a-3p was significantly downregulated in nonalcoholic fibrosing steatohepatitis and activated HSCs. Overexpression of miR-130a-3p contributed to the development of liver fibrosis by inhibiting proliferation, activation, matrix production, and the deposition of collagen, as well as inducing apoptosis in HSCs by suppressing the TGF-*β*/SMAD signaling pathway (Figure 8g). Further HSC apoptosis may lead to an eventual resolution of liver pathology. Therefore, miR-130a-3p might serve as a novel regulator in the pathogenesis of nonalcoholic fibrosing steatohepatitis.

## Materials and Methods

### Animal models of nonalcoholic fibrosing steatohepatitis

Eight-week-old male C57BL/6J mice were bred and housed as previously described.^23^ After 1 week of acclimation, the mice were divided into two groups (*n*=6 per group). The mice in the control group were fed a diet supplemented with choline bitartrate and dl-methionine (Research Diets, Inc., New Brunswick, NJ, USA), and the mice in the nonalcoholic fibrosing steatohepatitis group were fed the MCD diet (Research Diets). The mice were killed after 8 weeks, following a 12-h fast. Portions of the livers were either fixed in 10% formalin for histological analysis or snap-frozen in liquid nitrogen followed by storage at −80 °C in a freezer until use. All of the protocols and procedures were performed following the guidelines of the Hebei Committee for Care and Use of Laboratory Animals and were approved by the Animal Experimentation Ethics Committee of Hebei Medical University.

### Histological analysis and biochemical analysis

Hematoxylin and eosin (H&E) and Masson’s trichrome were used to stain the paraffin-embedded liver sections (5 *μ*m thick), which were scored for hepatic steatosis, inflammation, and fibrosis as described previously by the Brunt’s criteria and the histological scoring system for NAFLD issued by the Pathology Committee of the Nonalcoholic Steatohepatitis Clinical Research Network.^41^ Serum ALT and AST levels were measured using the enzymatic kinetic method with an automatic biochemical analyzer (Olympus AU2700, Tokyo, Japan) according to the manufacturer’s instructions.

### microRNA microarray assay

Total RNA was extracted from 20 mg of liver tissue from the MCD diet-fed mice (*n*=3 mice per group) and the control diet-fed mice (*n*=2 mice per group) using TRIzol reagent (Invitrogen, Carlsbad, CA, USA) according to the manufacturer’s instructions. The *μ*Paraflo MicroRNA Microarray Assay was performed using a service provider (LC Sciences, Houston, TX, USA). The assay started with the 3′-extension with a poly (A) tail of 4 to 8 *μ*g of total RNA using poly (A) polymerase. An oligonucleotide tag was then ligated to the poly (A) tail for later fluorescent dye staining. Hybridization was performed overnight on a *μ*Paraflo microfluidic chip using a micro-circulation pump (Atactic Technologies, Houston, TX, USA). After RNA hybridization, the tag-conjugating Cy3 dye was circulated through the microfluidic chip for dye staining. Fluorescence images were collected using GenePix 4000B Microarray Scanner Molecular Device, Sunnyvale, CA, USA and digitized using Array-Pro image analysis software (Media Cybernetics, Rockville, MD, USA). The data were analyzed by first subtracting the background and then normalizing the signals using a LOWESS filter (locally weighted regression).

### Immunohistochemistry and *in situ* hybridization

ISH was performed to detect the presence of miR-130a-3p in liver tissues using a digoxin-labeled oligonucleotide probe (5′-GCCCTTTTAACATTGCACTG-3′), as instructed by the protocol included with the ISH detection kit (BIO-HIGH Technology, Shijiazhuang, China). IHC for TGFBR1, TGFBR2, Col-1, and Col-4 (Abcam, Cambridge, MA, USA) was performed using paraffin-embedded liver sections. Negative controls were carried out by replacing the specific primary antibodies with PBS. The sections were incubated with a horseradish peroxidase (HRP)-conjugated secondary antibody and developed with diaminobenzidine tetrahydrochloride as a substrate (ZSGB Biotechnology, Beijing, China) for 1 min. The slides were then counterstained briefly with hematoxylin, washed in PBS, dehydrated in graded alcohol, and mounted for microscopic analysis.

### Patients with NAFLD-related liver fibrosis

Eight patients with NAFLD and six age- and gender-matched healthy subjects who were all from the Third Hospital of Hebei Medical University (Shijiazhuang, China) were included in this study. The demographic characteristics of the study subjects are listed in Table 1. NAFLD was confirmed using serology and liver biopsies. Normal liver tissues were obtained from liver transplant donors of donation after cardiac death (DCD) and written informed consent was obtained prior to sample collection. The study was approved by the Human Ethics Committee of the Third Hospital of Hebei Medical University. All liver biopsy specimens were processed for H&E staining, Masson’s trichrome staining, IHC staining, and ISH.

### qRT-PCR analysis

RNA was isolated from liver tissues and cells using Trizol (Invitrogen). The isolated RNA was then reverse transcribed into cDNA using reverse transcriptase with either miRNA-specific stem-loop primers (RiboBio, Guangzhou, China) or oligo dT primers (Thermo, Waltham, MA, USA). Differential qRT-PCR was performed on an ABI 7500 Real-Time PCR system (Applied Biosystems, Foster City, CA, USA) using SYBR Green master mix (CoWin Biotech, Beijing, China). The relative abundance of miRNA was normalized to the small nuclear RNA U6, and the expression levels of the genes were normalized to the endogenous reference gene glyceraldehyde phosphate dehydrogenase. The relative amounts of the miRNAs and genes were measured using the 2^-△△Ct^ method. All of the qRT-PCR reactions were conducted in triplicate, and the primers used for the qRT-PCR are shown in Table 2.

### Prediction of potential miRNA gene targets

All gene targets of the differentially expressed miRNAs were predicted using five databases including TargetScan, miRanda, miRDB, PITA, and RNA22. miRWalk2.0 was used to help develop the strategy for target gene prediction and the subsequent bioinformatics analysis. The predicted targets of the upregulated and downregulated miRNAs were then subjected to GO (www.geneontology.org) analysis to uncover the miRNA gene regulatory networks of the biological processes and molecular functions.^42^ Enrichment provided a measure of the significance of the function, and pathway analysis was also used to determine the significant pathways of the differential genes according to KEGG. Fisher’s exact test and the *χ*^2^-test were used to classify the GO category and the major pathway. The FDR was calculated to correct the *P*-value, and a *P*-value of <0.001 and an FDR of <0.01 were considered to be statistically significant.

### Cell cultures

The HSC-T6 and HEK293T cells were cultured in Dulbecco’s Modified Eagle medium (DMEM) supplemented with 10% fetal bovine serum (FBS), 100 U/ml penicillin, and 100 g/ml streptomycin. The HSC-T6 and HEK293T cells were maintained under a humidified atmosphere of 95% air and 5% CO_2_, and routinely subcultured using trypsin/ethylenediaminetetraacetic acid every 4–5 days.

### Transfection of HSCs with miR-130a-3p

The HSC-T6 cells were seeded at a density of 2 × 10^5^/ml, and the medium was then replaced with fresh DMEM medium without antibiotics. The cells were transfected with 50 nM of miR-130a-3p mimic or mimics control (RiboBio) using Lipofectamine 2000 (Invitrogen) for the gain and loss of miR-130a-3p function experiments, respectively. After culturing for 5 h with the transfection mix, the cell culture medium was replaced with DMEM supplemented with 10% FBS and antibiotics. At 48 h post transfection, the cells were harvested by mild trypsinization and washed in phosphate-buffered saline. All of the experiments were repeated in triplicate.

### RNA interference and transfection

The HSC-T6 cells were transfected with siRNA against either TGFBR1 or TGFBR2 and negative control siRNA (RiboBio). The HSC-T6 cells were transfected using Lipofectamine 2000 (Invitrogen, Life Technologies, Carlsbad, CA, USA), and qRT-PCR and western blotting were performed to evaluate the knockdown efficiency of the siRNA. The synthesized oligos are shown in Table 2.

### Cell proliferation assay

Cell proliferation was assessed using an MTT assay (Sigma-Aldrich, St. Louis, MO, USA). Briefly, the HSC-T6 cells were seeded at a density of 5 × 10^3^ cells per well in 96-well plates and transfected with miR-130a-3p mimics as described above. The cells were assayed for proliferation 24, 48, and 72 h later. After culturing, 5 mg/ml of MTT was added, and the cells were incubated at 37 °C for another 4 h. The medium was then replaced, and formazan crystals were dissolved in 150 *μ*l of dimethylsulfoxide. The OD was determined using a Thermomax microplate reader (Bio-Tek EL, Winooski, VT, USA) at a wavelength of 490 nm. The experiments were conducted three times independently.

### Western blot analysis

Liver tissue and cells were lysed in radioimmunoprecipitation buffer. Lysates were centrifuged at 14 000  × *g* for 10 min to recover the supernatant. About 80 *μ*g of sample proteins were separated by 10 or 12% sodium dodecyl sulfate-polyacrylamide gel electrophoresis gel and transferred onto PVDF membranes (Millipore Corporation, Billerica, MA, USA) by electroblotting. After being blocked for 60 min with buffer containing 0.1% Tween-20 and 5% milk, the membranes were incubated overnight at 4 °C with primary antibodies against *α*-SMA, Col-1, Col-4 (Bioss, Beijing, China), Smad2, Smad3 (Novus Biologicals, Novus Biologicals, Littleton, CO, USA), TGFBR1 (Abcam), TGFBR2 (Novus Biologicals), MMP-2, MMP-9 (ProteinTech Group, Chicago, IL, USA), TGF-*β*_1_, caspase-3, caspase-9, and PARP1 (Novus Biologicals, Littleton, CO, USA). After being washed, they were incubated with suitable HRP-conjugated secondary antibodies (ProteinTech Group) for 1 h at room temperature, and then were visualized by enhanced chemiluminescence method. *β*-actin (Boster, Wuhan, China) was served as a loading control. The intensity of each protein band of interest was quantified by densitometry using Quantity One 4.6.3 software (Bio-Rad, Hercules, CA, USA).

### Annexin V-FITC/PI apoptosis assay

For apoptosis analysis, quantification of apoptotic cells was performed with double fluorescence staining with APC Annexin V and PI (BD Biosciences, San Jose, CA, USA) with subsequent flow cytometry analysis according to the manufacturer’s instructions. The relative proportion of Annexin V-positive and PI-negative cells was determined using the ModFitLT software (Becton Dickinson, San Diego, CA, USA) and counted as early apoptotic cells (Annexin V-positive, PI-negative), late apoptotic cells (Annexin V-positive, PI-positive), necrotic cells (Annexin V-negative, PI-positive), and viables (Annexin V-negative, PI-negative). The experiments were conducted three times independently.

### Luciferase activity assay

The cDNA fragments corresponding to the WT or mutant (Mut) seed region of TGFBR1 and TGFBR2-3′-UTR were amplified with the Fusion RT-PCR from total DNA extracted from liver tissue. The PCR products were cloned into the designated multiple cloning sites downstream of the luciferase open reading frame between *Xho*I and *Not*I restriction sites of the psiCHECK-2 luciferase vector (Promega, Madison, WI, USA). An empty luciferase reporter vector was used as a negative control. Sequencing confirmed all constructs. HEK-293 T cells were cultured in 24-well plates and each well was transfected with 200 ng of the respective psi-CHECK2 3′-UTR constructs, and 50 nM miR-130a-3p mimics or mimics control, using Lipofectamine 2000 transfection reagent (Invitrogen), according to manufacturer’s protocol. After 5 h, OptiMEM (Invitrogen) transfection medium was replaced with DMEM supplemented with 10% FBS. Cells were harvested and assayed at 48 h post transfection using the Luciferase Assay System (Promega). The synthesized oligos were shown in Table 2.

### Statistical analysis

Data are expressed as mean±S.D. and based on at least three independent experiments. One-way analysis of variance (ANOVA) test and Student’s *t*-test were used for statistical analysis. One-way ANOVA with *post hoc* Geisser–Greenhouse correction was used when more than two groups are compared. Two-tailed *t*-test was used when two groups are compared. The statistical analysis was performed using SPSS 17.0 (IBM, Armonk, NY, USA). *P*<0.05 was considered to indicate a statistically significant difference.

## Figures and Tables

**Table 1:** Clinical characteristics of human subject

	**All**	**Healthy control**	**NAFLD (*F*=2, NAS=3–6)**	***P*****-value**
	***n*****=14**	***n*****=6**	***n*****=8**	
Age, years (mean±S.D.)	44.07±5.88	41.67±6.98	45.88±4.55	0.1958
Gender, male (*n*, %)	7 (50.00%)	2 (33.33%)	5 (62.50%)	0.5892
Serum ALT (U/l)	33.00±14.99	17.33±3.50	44.75±6.36	***0.0000***
Serum AST (U/l)	33.79±14.36	19.67±8.02	44.38±6.19	***0.0000***

Abbreviations: F, fibrosis score; NAFLD, nonalcoholic fatty liver disease; NAS, nonalcoholic steatohepatitis activity score.

Bold and italics numbers indicate significant differences as *P*-value <0.05

**Table 2:** Primers for quantitative real-time PCR analysis

**Gene**	**Length of production (bp)**	**Primers**
*TGFBR1*	78	F 5′-AAACTTGCTCTGTCCACGG-3′
		R 5′-AATGGCTGGCTTTCCTTG-3′
*TGFBR1-3′-UTR*	159	F 5′-CCCTCGAGGGATGCACACCAAAATCTGCCC-3′
		R 5′-ATTTGCGGCCGCTTTACGACTTGTTCTGCTGGGCTA-3′
*TGFBR1-3′-UTR mut*	159	F 5′-CCCTCGAGGGCATTCCACAGCGGGAAGGAATGATTGTAAATCA-3′
		R 5′-ATTTGCGGCCGCTTTATTCCTTCCCCAGGTGGAATGAAATCACAATGTC-3′
*TGFBR2*	133	F 5′-GTAATAGGACTGCCCATCCAC-3′
		R 5′-GATTTCTGGTTGTCACAGGTG-3′
*TGFBR2-3′-UTR*	191	F 5′-CCCTCGAGGGTGCCTTGGAGACTGTCATGG-3′
		R 5′-ATTTGCGGCCGCTTTATGTCTCCTTGCAGAGTATGGC-3′
*TGFBR2-3′-UTR mut*	191	F 5′-CCCTCGAGGGCCAATAACGTGGCGCGATTATTAATGCCTGTGTGT-3′
		R 5′-ATTTGCGGCCGCTTTAGGCATTAATAAGCCCGCCACGTTATTGGCTGTTGT-3′
*TGF-β1*	82	F 5′-GAGCCCTGGACACCAACTAT-3′
		R 5′-TCCTTGCGGAAGTCAATG-3′
*Smad2*	85	F 5′-AGCAGAATACCGAAGGCAG-3′
		R 5′-TTTGTCCAACCACTGTAGAGGT-3′
*Smad3*	61	F 5′-GCTGCTCTCCAATGTCAACAG-3′
		R 5′-TCTTCCGATGTGTCTCCGT-3′
*α-SMA*	98	F 5′-CTGACAGAGGCACCACTGAA-3′
		R 5′-CATCTCCAGAGTCCAGCACA-3′
*Col-1*	70	F 5′-GATTGAGAACATCCGCAGC-3′
		R 5′-CATCTTGAGGTCACGGCAT-3′
*Col-4*	107	F 5′-ATCTCTGCCAGGACCAAGTG-3′
		R 5′-CGGGCTGACATTCCACAAT-3′
*MMP-2*	103	F 5′-TTTCTATGGCTGCCCCAAGG-3′
		R 5′-GTCAAGGTCACCTGTCTGGG-3′
*MMP-9*	87	F 5′-AGAACCAATCTCACCGACAGG-3′
		R 5′-CGACTCTCCACGCATCTCT-3′
*Casp3*	185	R 5′-ACTGGACTGTGGCATTGAGAC-3
		R 5′-TTGTCGGCATACTGTTTCAGC-3
*Casp9*	75	R 5′-GCAGATTTGGCTTACATCCTG-3
		R 5′-ACGGCAGAAGTTCACATTGT-3
*PAPR1*	123	R 5′-CCGCATACTCCATCCTCAGT-3
		R 5′-GCTTCTTCATCCCAAAGTCG-3
*GAPDH*	112	F 5′-CAAGAAGGTGGTGAAGCAGG-3′
		R 5′-AAAGGTGGAGGAGTGGGTGT-3′
